# First true brackish-water nudibranch mollusc provides new insights for phylogeny and biogeography and reveals paedomorphosis-driven evolution

**DOI:** 10.1371/journal.pone.0192177

**Published:** 2018-03-14

**Authors:** Tatiana Korshunova, Kennet Lundin, Klas Malmberg, Bernard Picton, Alexander Martynov

**Affiliations:** 1 Koltzov Institute of Developmental Biology, Moscow, Russia; 2 Zoological Museum of the Moscow State University, Moscow, Russia; 3 Gothenburg Natural History museum, Gothenburg, Sweden; 4 Gothenburg Global Biodiversity Centre, Gothenburg, Sweden; 5 Aquatilis, Nostravägen 11, Gothenburg, Sweden; 6 National Museums Northern Ireland, Cultra, United Kingdom; Laboratoire de Biologie du Développement de Villefranche-sur-Mer, FRANCE

## Abstract

A unique example of brackish water fjord-related diversification of a new nudibranch genus and species *Bohuslania matsmichaeli* gen. n., sp. n. is presented. There are only few previously known brackish-water opisthobranchs and *B*. *matsmichaeli* gen. n., sp. n. is the first ever described brackish-water nudibranch with such an extremely limited known geographical range and apparently strict adherence to salinity levels lower than 20 per mille. Up to date the new taxon has been found only in a very restricted area in the Idefjord, bordering Sweden and Norway, but not in any other apparently suitable localities along the Swedish and Norwegian coasts. We also show in this study for the first time the molecular phylogenetic sister relationship between the newly discovered genus *Bohuslania* and the genus *Cuthona*. This supports the validity of the family Cuthonidae, which was re-established recently. Furthermore, it contributes to the understanding of the evolutionary patterns and classification of the whole group Nudibranchia. Molecular and morphological data indicate that brackish water speciation was triggered by paedomorphic evolution among aeolidacean nudibranchs at least two times independently. Thus, the present discovery of this new nudibranch genus contributes to several biological fields, including integration of molecular and morphological data as well as phylogenetic and biogeographical patterns.

## Introduction

Nudibranchs or sea slugs are an emerging model group in many different fields [1–4]. The molecular phylogeny of Nudibranchia, and particularly in the case of the aeolidacean nudibranchs is at present an actively debated evolutionary and taxonomic topic [5–7]. There is a recent suggestion based on a molecular phylogenetic analysis to merge a considerable part of the taxonomic diversity of aeolidacean nudibranchs into the single family Fionidae [8]. However, this suggestion was recently contested and rejected in the frame of a major reassessment of the aeolidacean nudibranchs [9]. The nudibranch molluscs are well known for being almost exclusively marine [1] and in the seas with low salinity (e.g. in the Black and Baltic Seas) their diversity declines considerably [10,11]. To date there are no specific studies which have utilized an integrative molecular and morphological approach to infer patterns of brackish water-related diversification in nudibranchs, but this has been considered in other opisthobranchs [12–14].

Habitat shifts are one of the fundamental aspects of biological evolution [15–18]. The interaction between Earth’s two major marine and fresh water realms forms a unique narrow, dynamic zone with lower salinity than in the oceans, commonly termed brackish water [19,20]. Such a zone occurs in estuarine areas [21,22] and in other transitory water bodies sometimes with limited exchange of oceanic water, like the Baltic Sea, or the inner part of fjords. Furthermore, since brackish waters repeatedly have been colonized by marine taxa, this often leads to intricate phylogenetic patterns, as has recently been revealed from several metazoan phyla [23,24]. Habitat shifts have played a fundamental role in the evolution of the pulmonate molluscs, which is the largest terrestrial metazoan group after insects and chordates, as they shifted from marine to terrestrial environments also through intermediate stages in intertidal and brackish waters [25].

Here we report a remarkable new genus and species living in a sheltered and stable brackish water environment adjacent to an estuary at the innermost part of the Idefjord, bordering Sweden and Norway. Up to date the new taxon was not found in any other apparently suitable localities on Swedish and Norwegian coasts but only in a very restricted area of the Idefjord. This is in strong contrast to the very few other currently known nudibranch species, which have been recorded from brackish water habitats, but which also have a cosmopolitan broad distribution in different oceans [26]. There are also a few more examples of brackish-water diversification (and even fresh water) in other opisthobranch groups (e.g. acochlidians–see [27]), but among nudibranchs so far, the present finding is the only example of strict adherence to a true brackish water habitat with extremely limited geographical distribution. This highlights the importance of these findings, both for general understanding of the reliability of a separate specific brackish water fauna and for conservational science.

**Fig 1:**
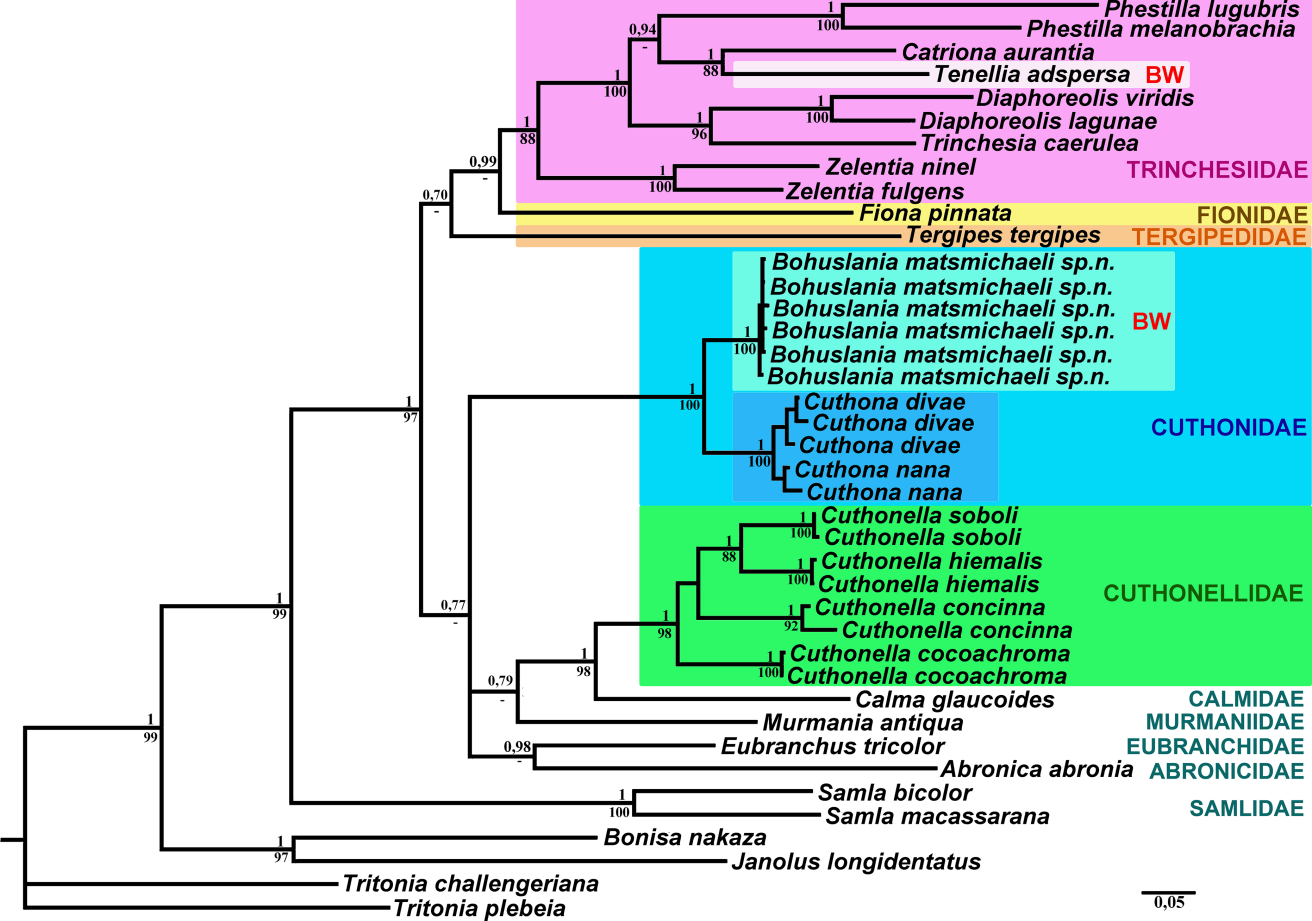
Phylogenetic tree of aeolidacean nudibranchs based on concatenated molecular data (COI + 16S + 28S + H3) represented by Bayesian Inference (BI). The aeolidacean families are highlighted. The brackish-water living, but non-related taxa *Bohuslania* gen. n. and *Tenellia* are indicated as “BW”. Numbers above branches represent posterior probabilities from Bayesian Inference. Numbers below branches indicate bootstrap values for Maximum Likelihood.

**Fig 2:**
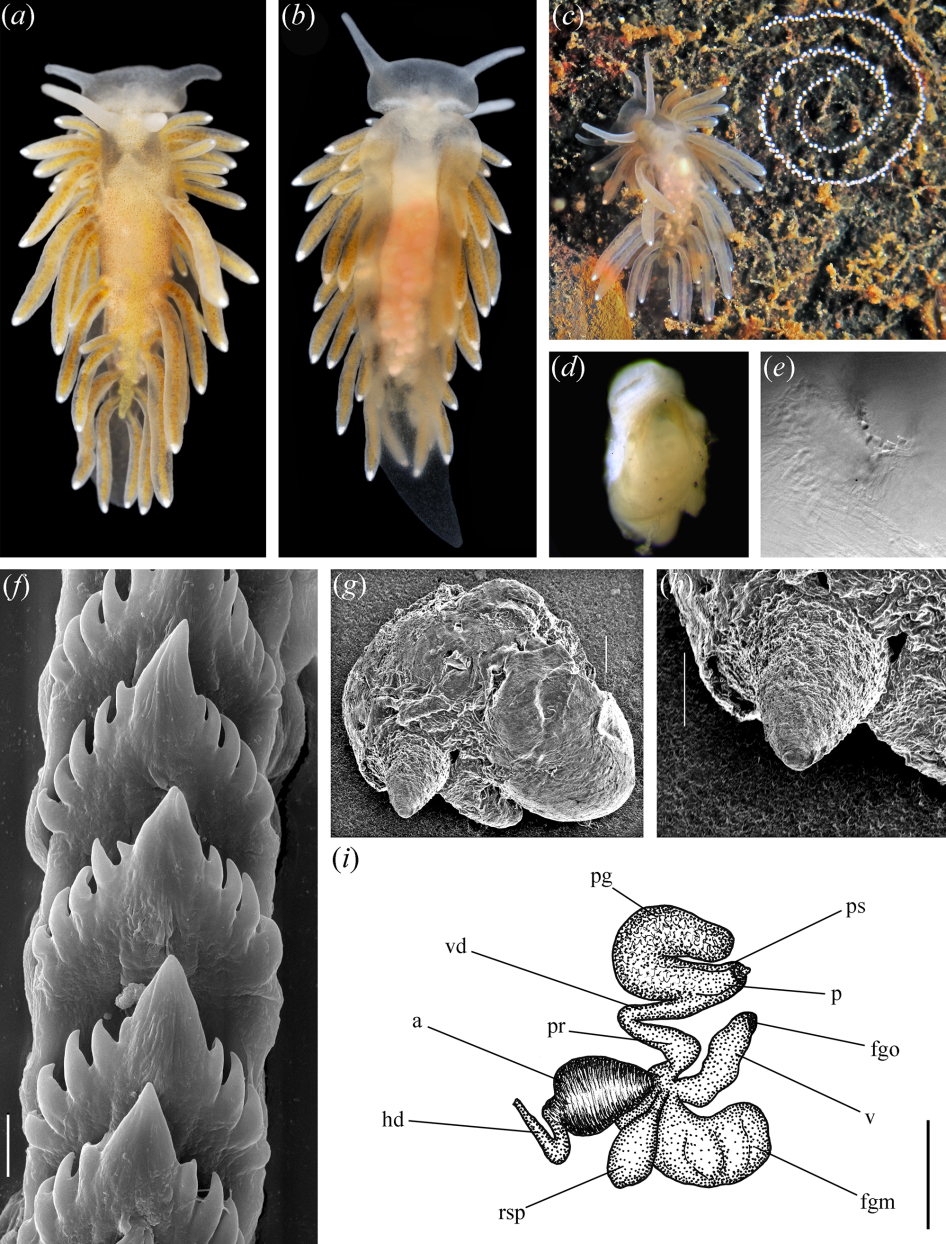
Morphology of *Bohuslania matsmichaeli* gen. n., sp. n. A, Dorsal appearance of the holotype; B. Ventral appearance holotype; C. Living specimen and its egg mass in situ; D, Pharynx and jaws (Paratype GNM 9024); E, Masticatory processes of jaws with denticles; F, Radula, central teeth (Holotype); G, Reproductive system of the holotype (non-destructive SEM with “nano-coating”); H, copulative organ (same technique as in G); I, Scheme of reproductive system. Scales: 10 μm (F, G, H), 500 μm (I). Abbreviations: a–ampulla, fgm–female gland mass, fgo–female opening, hd–hermaphroditic duct, p–penis, pg–penial gland, pr–prostate, ps–penial sheath, rsp–proximal receptaculum seminis, vd–vas deferens, v–vagina.

**Fig 3:**
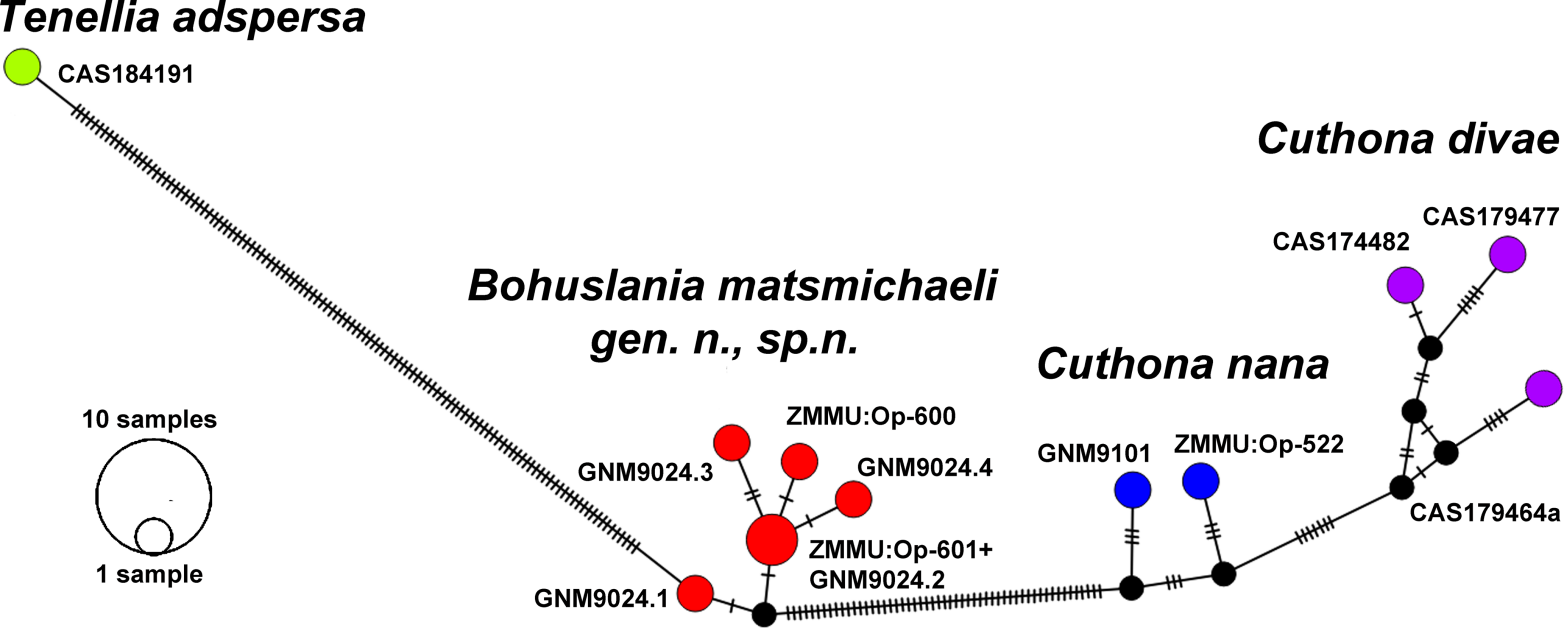
The haplotype network based on cytochrome c oxidase subunit I molecular data showing genetic mutations occurring within family Cuthonidae and brackish-waters aeolidacean nudibranch *Tenellia adspersa* of the family Trinchesiidae.

**Fig 4:**
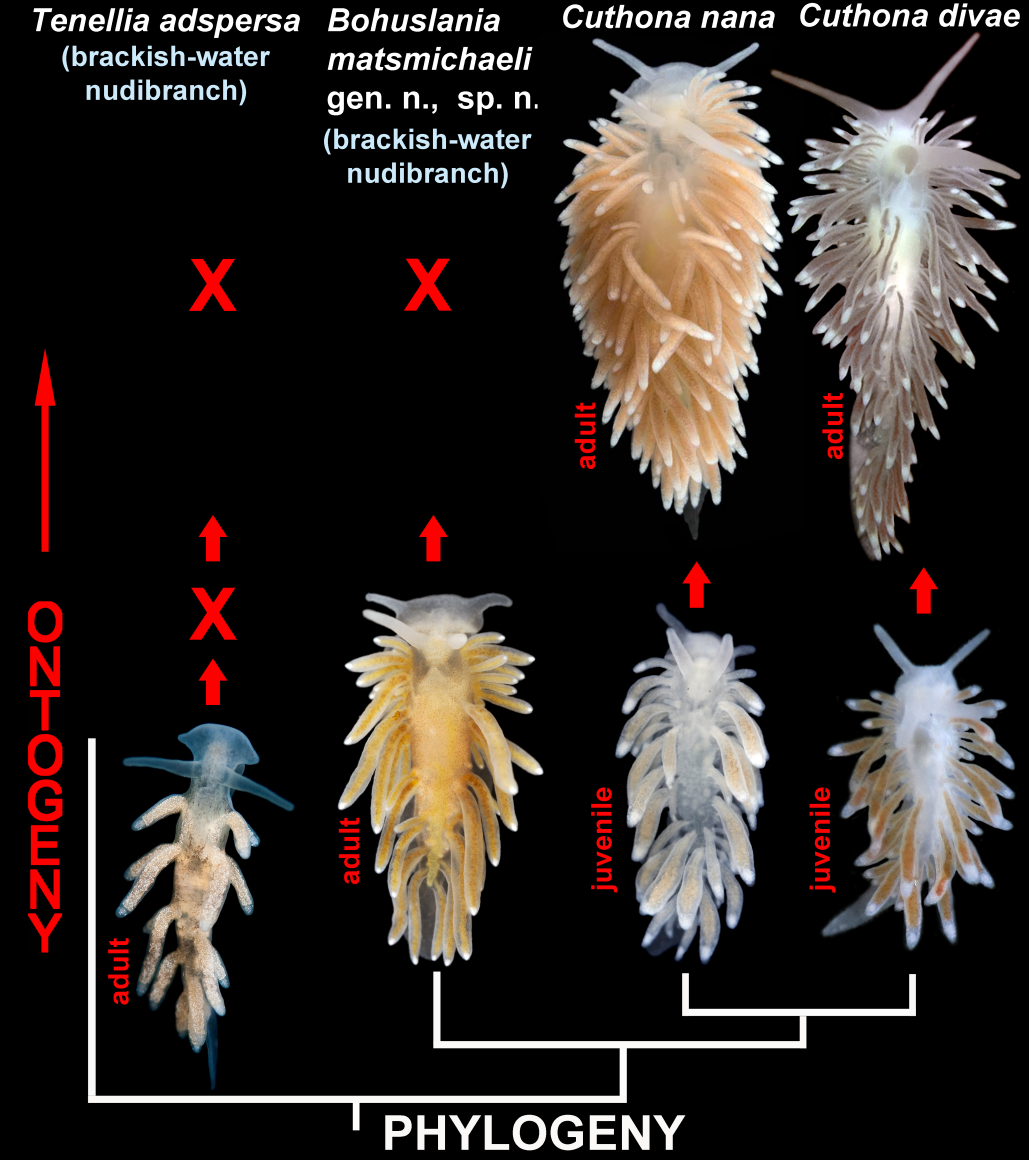
Ontogenetic and phylogenetic framework for evidence of parallel paedomorphosis driven-evolution within the brackish-water nudibranchs of the families Cuthonidae (*Bohuslania* gen. n.) and Trinchesiidae (*Tenellia adspersa*). Adults (14 and 20 mm) of marine species *Cuthona nana* and *C*. *divae* respectively, considerably differ from adults of brackish-water *Bohuslania matsmichaeli* gen. n., sp. n., whereas juveniles of *C*. *nana* (6 mm length) and *C*. *divae* (5 mm length) are similar to the adult of *B*. *matsmichaeli* (10 mm length) by presence of only 3–4 anterior ceratal rows and absence of numerous pre-rhinophoral digestive gland branches. Because *Bohuslania*, compared to *Cuthona*, does not develop further to reach the stage with numerous anterior ceratal rows, this stage is considered missing in *Bohuslania* (indicated by “X” on the scheme) due to heterochronic developmental shifts leading to the adult paedomorphic external morphology. The brackish-water species *Tenellia adspersa* (adult, 6 mm length) demonstrates a striking paedomorphic feature in presence of secondary oral veil. However, it belongs to the family Trinchesiidae which is more distantly related to the family Cuthonidae according to the present molecular phylogenetic analysis (Figs 1 and 2). Since the appearance of the small oral veil occurs in earlier ontogeny of aeolidacean nudibranchs and predates formation of oral tentacles and numerous anterior ceratal rows, the two stages of development of external features are considered as missing in *Tenellia adspersa* (indicated by two “X” on the scheme).

Furthermore, based on integrative molecular and morphological evidence, it is shown that a brackish water-related evolutionary diversification has occurred within different aeolidacean nudibranch clades in parallel, including the newly discovered genus *Bohuslania*, by underlying paedomorphosis-driven developmental changes (Figs 1–4). Thus, the discovery of the new unique brackish water taxon in a specific Scandinavian fjord suggests broad implications and it influences our general understanding of the importance of habitat shifts, broad-scale diversification patterns and considerably influences phylogeny and classification of the whole nudibranch group Aeolidacea.

## Materials and methods

### Sample data

A total of seven specimens of the new genus were collected in the Idefjord in Sweden. The outer half of the fjord runs in a southwesterly to northeasterly direction from the fjord opening to the Norwegian city of Halden and the outlet of the Tista river. Here the fjord makes a bend in straight angle and runs in a southeasterly direction, with the Enningdal river estuary at the innermost end. Samples were collected at the midsection of the inner part of the fjord using Scuba diving at 5–7 m depth by Mats Larsson and Michael Lundin in October 2013, March 2014 and September 2015. At this location and depth, the salinity varies from 15 to 18 per mille [28]. Eight species of aeolidacean nudibranchs from several families were collected at Väderö Islands in Sweden, in the North Atlantic near Gulen Dive Resort in Norway, at Banyuls-sur-Mer in France, and in the White Sea and in the Sea of Japan in Russia using Scuba diving. No special permission was needed for collection of nudibranch molluscs in any of these areas. All specimens were deposited in the Gothenburg Natural History Museum (GNM) and the Zoological Museum of Moscow State University (ZMMU).

### Morphological analysis

The external and internal morphology was studied under a stereomicroscope and using full frame digital cameras, a Nikon D-810 and a Nikon D300 with Nikon 60mm lens and 2.0 Kenko converter. For the description of internal features, both preserved and fresh specimens (when available) were dissected under the stereomicroscope. The buccal mass of each specimen was extracted and soaked in 10% sodium hypochlorite solution to dissolve connective and muscle tissues, leaving only the radula and the jaws. The features of the jaws of each species where analysed under both stereomicroscope and scanning electron microscope, and then drawn. The coated radulae were examined and photographed using a scanning electron microscope (CamScan). The reproductive systems of the different species were also examined and drawn using the stereomicroscope.

A novel method of non-destructive scanning electron microscopy is applied for the first time to study nudibranch molluscs. The method implies use of the scanning electron microscope but without drying of the studied object. The samples were prepared using the amphiphilic surfactant compound polyoxyethylene sorbitan monolaurate (Tween 20) and by following the method described in [29, 30]. AM and TK have performed the non-destructive SEM study of the reproductive system of *Bohuslania matsmichaeli* gen. n., sp. n. by using a special dual scanning electron and light microscopy system Keyence VHX-D510, at the National Museum of Nature and Science, Tsukuba, Japan.

### Taxon sampling for molecular analysis

For comprehensive aeolidacean taxon sampling twenty one species of the aeolidacean nudibranchs from the genera *Abronica*, *Calma*, *Cuthona*, *Cuthonella*, *Diaphoreolis*, *Eubranchus*, *Fiona*, *Murmania*, *Phestilla*, *Tenellia*, *Tergipes*, *Zelentia* and six outgroup species from the genera *Bonisa*, *Janolus*, *Samla*, *Tritonia* were used. To analyze the position of the new genus and species *Bohuslania matsmichaeli* gen. n., sp. n. taxa which previously were included into the genus *Cuthona* s.l. and related taxa were specifically included.

Currently the genus *Cuthona* is considerably restricted to only three species [7, 9], two of which, *Cuthona nana* (Alder & Hancock, 1842) from northern Atlantic and *C*. *divae* (Er. Marcus, 1961) from northeastern Pacific were used for the analysis. A third species (*Cuthona hermitophila* Martynov, Sanamyan & Korshunova, 2015) inhabits the northwestern Pacific (the Sea of Japan) [31,32]. There are molecular data for a *Cuthona* species from the Russian part of the Sea of Japan in the GenBank [33]. However, we were not able to use these data because the results are misleading. Specifically, the accession number KU133321 which is indicated in [33] as “*Cuthona nana* from Netherlands” is Human herpesvirus 2 strain G DNA polymerase (UL30) gene. Furthermore, a species which is indicated in [33] as *Cuthona nana* from the Barents Sea under accession number KU133317 (voucher number ie112) after blast checking turned out to be the completely different taxon *Cuthonella concinna*. *Cuthona nana* KU133315 that is claimed to be collected from the Sea of Japan was actually collected in the Barents Sea according to the GenBank voucher number “ie76”. Therefore, we were not able to use such confused data in our analysis and it was impossible to repeat the tree that was presented in [33]. We therefore do not trust any data on “*Cuthona*” or “*Cuthonella*” that were sequenced in [33].

To compare the new genus and species with the only previously known brackish water aeolidacean taxon *Tenellia adspersa* (Nordmann, 1845) it was also included (see Table 1 for full list of samples, localities, and voucher references).

**Table 1:** List of samples, localities, and voucher references.

Species name	Voucher	Locality	COI	16S	H3	28S
*Abronica abronia* (MacFarland, 1966)	CAS181319	California	KY128919	KY128716	KY128508	-
*Bohuslania matsmichaeli* sp.n.	ZMMU:Op-600	Sweden, Ide fiord	**MG323542**	**MG323548**	**MG323563**	**MG323554**
*Bohuslania matsmichaeli* sp.n.	ZMMU:Op-601	Sweden, Ide fiord	**MG323541**	**MG323547**	**MG323562**	**MG323553**
*Bohuslania matsmichaeli* sp.n.	GNM9024.1	Sweden, Ide fiord	**MG323537**	-	**MG323558**	-
*Bohuslania matsmichaeli* sp.n.	GNM9024.2	Sweden, Ide fiord	**MG323538**	-	**MG323559**	-
*Bohuslania matsmichaeli* sp.n.	GNM9024.3	Sweden, Ide fiord	**MG323539**	-	**MG323560**	-
*Bohuslania matsmichaeli* sp.n.	GNM9024.4	Sweden, Ide fiord	**MG323540**	-	**MG323561**	-
*Bonisa nakaza* Gosliner, 1981	CASIZ176146	South Africa	HM162746	HM162670	HM162579	-
*Calma glaucoides* (Alder & Hancock, 1854)	ZMMU:Op-603	Norway, Gulen	**MG323544**	**MG323550**	MG323565	-
*Catriona aurantia* (Alder & Hancock, 1842)	ZMMU:Op-545	Norway, Gulen	KY985467	MF523458	**MG386404**	MF523524
*Cuthona divae* (Er. Marcus, 1961)	CAS174482	California	KY128938	KY128733	KY128526	-
*Cuthona divae* (Er. Marcus, 1961)	CAS179464a	California	KY128940	KY128735	KY128528	-
*Cuthona divae* (Er. Marcus, 1961)	CAS179477	California	KY128944	KY128739	KY128532	-
*Cuthona nana* (Alder & Hancock, 1842)	ZMMU:Op-522	Russia, Barents Sea	MF523376	MF523397	MF523301	MF523473
*Cuthona nana* (Alder & Hancock, 1842)	GNM9101	Sweden, Gullmar fiord	KY128915	KY128708	KY128501	-
*Cuthonella cocoachroma* (Williams & Gosliner, 1979)	CAS179471	California	KY128925	KY128720	KY128513	-
*Cuthonella cocoachroma* (Williams & Gosliner, 1979)	CAS185193	California	KY128931	KY128726	KY128519	-
*Cuthonella concinna* (Alder & Hancock, 1843)	ZMMU:Op-523	Russia, White Sea	MF523377	MF523459	MF523302	MF523525
*Cuthonella concinna* (Alder & Hancock, 1843)	GNM8866	Sweden, Vadero Islands	**MG323543**	**MG323549**	MG323564	**MG323555**
*Cuthonella hiemalis* (Roginskaya, 1987)	ZMMU:Op-186	Russia, White Sea	**MG323545**	**MG323551**	-	**MG323556**
*Cuthonella hiemalis* (Roginskaya, 1987)	WS3439	Russia, White Sea	KY129008	KY128800	KY128596	-
*Cuthonella soboli* Martynov, 1992	ZMMU:Op-524	Russia, Japan Sea	MF523378	MF523457	MF523303	MF523523
*Cuthonella soboli* Martynov, 1992	ZMMU:Op-604	Russia, Japan Sea	**MG323546**	**MG323552**	MG323566	**MG323557**
*Diaphoreolis lagunae* (O'Donoghue, 1926)	CAS179465a	California	KY128956	KY128749	KY128543	-
*Diaphoreolis viridis* (Forbes, 1840)	ZMMU:Op-537	Russia, White Sea	**MG266028**	**MG266026**	**MG266029**	**MG266027**
*Eubranchus tricolor* Forbes, 1838	ZMMU:Op-525	Norway, Gulen	MF523379	MF523399	MF523304	MF523475
*Fiona pinnata* (Eschscholtz, 1831)	MCNCN/ADN51997	Morocco	JX087558	JX087492	JX087628	-
*Janolus longidentatus* Gosliner, 1981	CASIZ176320	South Africa	HM162749	HM162673	HM162582	-
*Murmania antiqua* Martynov, 2006	ZMMU:Op-399	Russia, Kara Sea	MF523390	MF523394	MF523315	MF523470
*Phestilla lugubris* (Bergh, 1870)	CAS177437	Philippines	KY129075	KY128866	KY128660	-
*Phestilla melanobrachia* Bergh, 1874	CAS177299	Philippines	KY129079	KY128870	KY128664	-
*Samla bicolor* (Kelaart, 1858)	ZMMU:Op-68	Vietnam, Nha Trang	MF523383	MF523436	MF523308	MF523503
*Samla macassarana* (Bergh, 1905)	CAS181283	Philippines	KY129059	KY128850	KY128644	-
*Tenellia adspersa* (Nordmann, 1845)	CAS184191	New Hampshire	KY129085	KY128876	KY128668	-
*Tergipes tergipes* (Forsskål in Niebuhr, 1775)	WS3463	Barents Sea	KY129090	KY128881	KY128673	-
*Trinchesia caerulea* (Montagu, 1804)	ZMMU:Op-622	Norway, Gulen	**MG266024**	**MG266022**	**MG266025**	**MG266023**
*Tritonia challengeriana* Bergh, 1884	CASIZ171177	Bouvet Island	HM162718	HM162643	HM162550	-
*Tritonia plebeia* Johnston, 1828	ZMMU:Op-572	Norway	KX788134	KX788122	-	KX788132
*Zelentia fulgens* (MacFarland, 1966)	CAS185194	California	KY128952	KY128747	KY128540	-
*Zelentia ninel* Korshunova, Martynov & Picton, 2017	ZMMU:Op-509	Russia, Barents Sea	KY952178	MF523400	MF523242	MF523476

### Molecular analysis

Small pieces of tissue were used for DNA extraction with Diatom™ DNA Prep 100 kit by Isogene Lab, according to the producer’s protocols. Extracted DNA was used as a template for the amplification of partial sequences of the COI, 16S, H3 and 28S (see Table 2 for the primers). Polymerase chain reaction (PCR) amplifications were carried out in a 20-μL reaction volume, which included 4 μL of 5x Screen Mix (Eurogen Lab), 0.5 μL of each primer (10 μM stock), 1 μL of genomic DNA, and 14 μL of sterile water. The amplification of COI was performed with an initial denaturation for 1 min at 95°C, followed by 35 cycles of 15 sec at 95°C (denaturation), 15 sec at 45°C (annealing temperature), and 30 sec at 72°C, with a final extension of 7 min at 72°C. The 16S amplification began with an initial denaturation for 1 min at 95°C, followed by 40 cycles of 15 sec at 95°C (denaturation), 15 sec at 52°C (annealing temperature), and 30 sec at 72°C, with a final extension of 7 min at 72°C. The amplification of H3 and 28S began with an initial denaturation for 1 min at 95°C, followed by 40 cycles of 15 s at 95°C (denaturation), 15 s at 50°C (annealing temperature) and 30 s at 72°C, with a final extension of 7 min at 72°C. DNA sequences of both strands were obtained using the ABI PRISM® BigDye™ Terminator v. 3.1. on an automated DNA sequencer (Applied Biosystems Prism 3700). Some COI sequences were produced at the Canadian Centre for DNA Barcoding (CCDB), using their automated systems for extraction, PCR and sequencing.

**Table 2:** Primers.

Name	5′→3′	References
LCO 1490	GGTCAACAAATCATAAAGATATTGG	[81]
HCO 2198	TAAACTTCAGGGTGACCAAAAAATCA	[81]
16S arL	CGCCTGTTTAACAAAAACAT	[82]
16S R	CCGRTYTGAACTCAGCTCACG	[83]
H3 AF	ATGGCTCGTACCAAGCAGACGG	[84]
H3 AR	ATATCCTTGGGCATGATGGTGAC	[84]
28S C1	ACCCGCTGAATTTAAGCAT	[85]
28S C2	TGAACTCTCTCTTCAAAGTTCTTTTC	[86]

A total of 13 specimens were successfully sequenced for the mitochondrial genes cytochrome oxidase subunit I (COI) and 16S rRNA, and the nuclear genes Histone 3 (H3) and 28S rRNA (C1–C2 domain). Of these, COI and H3 from four specimens were sequenced in Gothenburg, the others in Moscow. Additional molecular data for 28 specimens of nudibranchs were obtained from GenBank (see Table 1; all new sequences highlighted in bold). Protein-coding sequences were translated into amino acids for confirmation of the alignment. All sequences were deposited in GenBank (Table 1, highlighted in bold). Original data and publicly available sequences were aligned with the MUSCLE algorithm [34]. Separate analyses were conducted for COI (657 bp), 16S (429 bp), H3 (327 bp) and 28S (337 bp). Gblocks 0.91b [35] was applied to discard poorly aligned regions for the 16S data set (using less stringent options; 10% of the positions were eliminated). An additional analysis was performed with all four concatenated markers (1750 bp). Evolutionary models for each data set were selected using MrModelTest 2.3 [36] under the Akaike information criterion [37]. Two different phylogenetic methods, Bayesian inference (BI) and Maximum Likelihood (ML), were used to infer evolutionary relationships. Bayesian estimation of posterior probability was performed in MrBayes 3.2 [38]. Four Markov chains were sampled at intervals of 1,000 generations. Analysis was started with random starting trees and 10^7^ generations. ML analysis was performed using RAxML 7.2.8 [39] with 1000 bootstrap replicates. Final phylogenetic tree images were rendered in FigTree 1.4.2. Nodes in the phylogenetic trees with Bayesian posterior values ≥0.96% and bootstrap values ≥90% were considered ‘highly’ supported, nodes with 0.90–0.95% and 80–89% accordingly were considered ‘moderately’ supported (lower support values were considered not significant).

To evaluate the genetic distribution of the different haplotypes, a haplotype network for the COI molecular data was reconstructed using Population Analysis with Reticulate Trees (PopART, http://popart.otago.ac.nz) with the TCS network method. The program Mega7 [40] was used to calculate the minimum uncorrected *p*-distances between all the sequences. Intra- and intergroup genetic distances were also examined.

Additionally, Automatic Barcode Gap Discovery (ABGD) [41] was used to define species. Alignment from the COI marker for *Bohuslania*, *Cuthona*, and *Tenellia* specimens were submitted and processed in ABGD using the Jukes-Cantor (JC69) and Kimura (K80) models and the following settings: a prior for the maximum value of intraspecific divergence between 0.001 and 0.1, 30 recursive steps.

### Nomenclatural acts

The electronic edition of this article conforms to the requirements of the amended International Code of Zoological Nomenclature, and hence the new names contained herein are available under that Code from the electronic edition of this article. This published work and the nomenclatural acts it contains have been registered in ZooBank, the online registration system for the ICZN. The ZooBank LSIDs (Life Science Identifiers) can be resolved and the associated information viewed through any standard web browser by appending the LSID to the prefix "http://zoobank.org/". The LSID for this publication is: urn:lsid:zoobank.org:pub:440744B1-789B-441F-BF78-E856182419A5. The electronic edition of this work was published in a journal with an ISSN,and has been archived and is available from the following digital repositories: PubMed Central, LOCKSS.

## Results

### Molecular phylogenetic relationships between *Bohuslania matsmichaeli* gen. n., sp. n. and other aeolidacean nudibranchs

The phylogenetic analysis was performed using six specimens of *Bohuslania matsmichaeli* gen. n., sp. n., twentyone species of aeolidacean nudibranchs (including *Cuthona nana*, *Cuthona divae*, several taxa which were until recently included in the genus *Cuthona* s.l., and several related taxa), and six outgroup specimens. The dataset consisted of one hundred and thirty nucleotide sequences. The combined dataset yielded a sequence alignment of 1750 positions. The GTR + I + G model was chosen for the combined dataset. The resulting concatenated tree (Fig 1) provided better resolution than COI, 16S, 28S or H3 separately. Bayesian Inference (BI) and Maximum Likelihood (ML) analyses based on the combined dataset for the mitochondrial COI and 16S, and the nuclear H3 and 28S genes yielded similar results (Fig 1).

The molecular phylogenetic analysis (Fig 1) supported the presence of a new species, *B*. *matsmichaeli* gen. n., sp. n. and inferred its phylogenetic position. All six *B*. *matsmichaeli* gen. n., sp. n. clustered together (PP = 1, BS = 100%) in a clade that is sister to the *Cuthona* clade and was found to be a highly supported lineage (PP = 1, BS = 100%). *Cuthona nana* and *C*. *divae* form two separate sister clades within the *Cuthona* clade, which is also a highly supported lineage (PP = 1, BS = 100%).

To define species, we use an integrative approach [9, 42] including phylogenetic tree topologies, ABGD analysis, pairwise distances as well as the haplotype network for the COI molecular data reconstructed using the Population Analysis with Reticulate Trees (PopART).

Regarding the supposedly fast-evolving COI marker, genetic distance values within the group *Bohuslania* gen. n. are 0.3%, within the group *Cuthona* s.str. are 2.25%, whereas distances between these groups are 9.2%. Intragroup distances within the *C*. *nana* group are 1.37%, and within the *C*. *divae* group are 1.42% versus intergroup distance of 2.82%. Intergroup distances between *B*. *matsmichaeli* gen. n., sp. n. group and the *C*. *nana* and *C*. *divae* clade are 9.2% and 9.5% respectively.

Minimum uncorrected *p*-distances of the COI marker which separate the holotype *B*. *matsmichaeli* gen. n. sp. n. from *C*. *nana* are 9.13% and from *C*. *divae* are 9.28%. From the other brackish waters aeolidacean nudibranch *Tenellia adspersa* there is a high genetic divergence of 18.87%. The ABGD analysis of the COI data set run with two different models revealed four potential species: *Bohuslania matsmichaeli* sp. n., *Cuthona nana*, *Cuthona divae*, and *Tenellia adspersa*. Results obtained by PopART showed a network of haplotypes that clearly clustered into four groups coincident with *B*. *matsmichaeli* gen. n., sp. n., *C*. *nana*, *C*. *divae* and brackish waters *T*. *adspersa*. (Fig 3).

The molecular phylogenetic results confirm the morphological analysis data. From the only other genus of the family Cuthonidae, *Cuthona* Alder & Hancock, 1855, *Bohuslania* gen. n. is morphologically readily distinguished by absence of the pre-rhinophoral rows of cerata and considerably smaller number of rows of the anterior digestive gland.

#### Taxonomy

- Class Gastropoda
- Order Nudibranchia
- Family Cuthonidae Odhner, 1934

#### Diagnosis

Body wide. Notal edges fully reduced. Cerata non-elevated, numerous per row. Ceratal rows branched. Anus acleioproctic or cleioproctic. Radula formula 0.1.0. Central teeth with strong cusp not compressed by adjacent lateral denticles. Vas deferens short, with weak prostate. Supplementary gland present, inserts to unarmed copulative organ.

#### Included genera

*Cuthona* Alder & Hancock, 1855, ***Bohuslania* gen. n.**

### Genus *Bohuslania* gen. n.

Type species: ***Bohuslania matsmichaeli* gen. n., sp. n.**

Urn:lsid:zoobank.org:act: lsid:zoobank.org:act:697C41ED-5B04-4CF2-B4C6-497062EF713D

#### Diagnosis

Three to four anterior rows of cerata, pre-rhinophoral cerata absent, head broad, oral tentacles placed towards lateral edges of head, no anterior foot corners, anus acleioproctic, jaws with single row of simple denticles, radular teeth narrow with prominent cusp, penis without stylet.

#### Etymology

After Bohuslän region in southern Sweden, where the only locality of this new genus and species in the Idefjord is known.

#### Species included

*B*. *matsmichaeli* sp. n.

### *Bohuslania matsmichaeli* sp. n.

(Figs 1–4)

#### Holotype

Sweden, Idefjord, 59° 02.400' N 11° 24.430'E, inner part of the Idefjord, off beach east of Boråsgården, 7 m depth, 2013-10-8, coll. Mats Larsson and Michael Lundin (ZMMU Op-600, 3 mm in length preserved, 10 mm living length).

#### Paratypes

Same locality and collectors as holotype, 5–7 m depth, 2013-10-8, 1 specimen (ZMMU Op-601, 3.5 mm in length preserved), 2014-03-26, 1 specimen (ZMMU Op-602, 3 mm in length preserved), 2015-09-18, coll. by Mats Larsson. 4 specimens (GNM Gastropoda 9024:1–4, 3–4 mm length preserved).

#### Type locality

Idefjord, Bohuslän region.

#### Etymology

This species is named in honour of Mats Larsson and Michael Lundin, who were the first to discover this unique taxon.

#### ZooBank registration

lsid:zoobank.org:act:2C02FDDF-E0EF-4DC7-9D28-087BA07696C6.

#### Description

The length of the preserved holotype is 3 mm (living is 10 mm). The preserved length of 9 mature paratype specimens ranged from 3 to 4 mm. The body is moderately broad (Fig 2*A* and 2*B*). The rhinophores are slightly longer than oral tentacles, smooth. The cerata are relatively long, thin and finger-shaped. Pre-rhinophoral cerata absent. Ceratal formula of the holotype: right (4,5,5; Anus,4,3,3,2,2) left (3,4,5; 4,3,2,2). Paratype specimens possess 3–4 pre-anal branches of anterior digestive gland. The head is broad, semicircular, oral tentacles placed towards edges of the head. The foot is moderate, anteriorly rounded, no foot corners.

#### Colour

The ground colour is semitranslucent sandy yellow with brownish hue (Fig 2*A*). The digestive branches in the cerata are light yellow to light greenish brown with small darker spots inside. Very fine darker spots of brown pigment are scattered over dorsal part of the body. The ovaries and eggs are visible through the body wall as pale white specks with a faint pink colour. Brightly white small cnidosacs shine through tops of the cerata.

#### Anatomy

*Digestive system*. The jaws are ovoid (Fig 2*D*). The masticatory processes of jaws bear a single row of simple conical denticles (Fig 2*E*). The radular formula in two studied specimens (5–6 mm preserved in length) is 20–23 x 0.1.0. The radular teeth are almost colourless. The central tooth is moderate, elongated, with prominent cusp and four to seven lateral denticles (Fig 2*F*).

*Reproductive system*. (Fig 2*G* and 2*I*). The ampulla is moderately large and swollen (Fig 2*I*, a). The prostate (Fig 2*I*, pr) is a widened proximal portion of vas deferens (Fig 2*I*, vd). The vas deferens transits to a penial sheath (Fig 2*I*, ps), which contains a conical penis without a stylet (Fig 2*H and* 2*I*, p). A massive bent supplementary (“penial”) gland inserts into base of the penis (Fig 2*I*, pg). The proximal receptaculum seminis is large, elongated-oval reservoir, on a stalk (Fig 2*I*, rsp). The vagina is relatively long and slightly convoluted (Fig 2*I*, v). The female part includes mucous and capsular glands (Fig 2*I*, fgm).

#### Biology

Inhabits mixed environment with stones and mud in shallow (5–7 m) brackish water (15–18 per mille). The athecate hydroid *Cordylophora caspia* was found in the area and can be a probable food source for *B*. *matsmichaeli*. Potentially two techate hydroids could possibly also inhabit the area, *Gonothyraea loveni* and *Laomedea flexuosa* but these were not found together with *B*. *matsmichaeli*. The egg mass is in the shape of a narrow whitish spiral cord with about 3 whorls (Fig 2*C*). The egg mass contains about 250 eggs.

#### Distribution

To date known only from the internal parts of the Idefjord in the Bohuslän region.

## Discussion

### The phylogenetic position of *Bohuslania*

In the present study we, for the first time, report the discovery of a genus that is robustly confirmed as a sister taxon to the genus *Cuthona* according to our molecular phylogenetic analysis (Fig 1). The combination of the molecular data, external and internal morphological data support the presence of a new nudibranch genus and species *Bohuslania matsmichaeli* gen. n., sp. n. and allow it to be placed in a broad-scope framework of the nudibranch phylogeny with novel implications for evolution, classification and biogeography of aeolidacean nudibranchs. The discovery of this new taxon considerably strengthens the re-establishment of the family Cuthonidae, since morphologically *Bohuslania* gen. n. is consistent with the diagnosis of the family Cuthonidae (a broad head with prominent lateral lobes, several rows of digestive glands, a supplementary gland inserted into the base of an unarmed copulative apparatus), but at the same time it possesses own apomorphies (lateral instead of central position of oral tentacles, fewer number of rows of anterior digestive gland, absence of pre-rhinophoral ceratal rows, invariably acleioproctic anus) which clearly distinguishes the genus from the genus *Cuthona*. In all molecular analyses that were performed in the course of the present study, *Bohuslania* was always placed as sister taxon to *Cuthona*. Recently a molecular phylogenetic study [8] controversially suggested that several morphologically very disparate taxa of aeolidacean nudibranchs should be united under the single family Fionidae. Such a decision is problematic since morphologically well supported families form separate molecular clades [7, 9; present study, Fig 1] and the lumping concept of the family Fionidae proposed by Cella et al. [8] lacks support by any morphological synapomorphies. After adding the novel molecular data on *Bohuslania*, the several distinct clades corresponding to the family groups among the traditional “tergipedids” (to which appropriate morphological apomorphies can be provided) remained stable (Fig 1). This confirms the necessity of the general re-classification of the aeolidacean nudibranchs based on integration of molecular and morphological evidences [7, 9]. Thus, the discovery of the molecularly-proven independent development-driven evolution in two distinct aeolidacean clades contributes to the vast field of habitat shift studies [15–18, 25] and to the taxonomy and phylogeny of one of the most intriguing invertebrate groups–the aeolidacean nudibranch molluscs [5, 7–9, 43–51].

### Biogeographic pattern of the new nudibranch taxon

Brackish-water adapted invertebrate species commonly demonstrate broad geographical ranges, which are often related to their considerable invasive ability when introduced to similarly brackish areas, such as harbours and rivermouths [52]. There are several brackish water invertebrates which are common in Scandinavian fjords and particularly in the Swedish Idefjord, including cnidarian hydroid polyp *Cordylophora caspia* (Pallas, 1771), nemertean *Cyanophthalma obscura* (Schultze, 1851), polychaete *Hediste diversicolor* (O.F. Müller, 1776), crustaceans *Neomysis integer* (Leach, 1814), *Gammarus zaddachi* Sexton, 1912, *Gammarus duebeni* Lilljeborg, 1852 and *Palaemon varians* Leach, 1813 [53–60] and the bryozoan *Einhornia crustulenta* (Pallas, 1766) [61]. Some invasive brackish-water species present in the inner part of the Idefjord are the gastropod mollusc *Potamopyrgus antipodarum* (Gray, 1843) [62] and the polychaete *Marenzelleria viridis* (Verrill, 1873) [63]. However, all these species from very different phyla have broad distribution at least in Atlantic and Mediterranean regions, and no one has been proved to be an extremely narrow endemic of a Swedish fjord. Sometimes there are rare species with limited distribution, an example is the ditch shrimp *Palaemon varians*, which along the Swedish coast is only found in a single pond, in which it occurs in large numbers. *P*. *varians* is, however, common along the shores of west Europe and its range reaches also the Mediterranean Sea [54]. The case of the geographically very restricted *Bohuslania* in the Idefjord stands in a strong contrast to this pattern. To date only a single aeolidacean nudibranch–*Tenellia adspersa*–is known to be a predominantly brackish-water species [26, 64]. However, *T*. *adspersa* is a cosmopolitan species and has also been found in waters with normal marine salinity [12, 26]. In addition, two other nudibranch species have been reported sometimes from the estuarine zone, *Corambe obscura* (Verrill, 1870), and *Trinchesia perca* (Er. Marcus, 1958); however, these species were only occasionally found in brackish waters, and have very broad distribution ranges including the normal oceanic environment [11]. Thus, *Bohuslania* is the first true brackish-water nudibranch, which does not occur in waters with salinity higher than 20 ‰, which also has an extremely restricted geographic range. The Idefjord, the only known habitat of the new brackish-water taxon, is a silled fjord. The sills inhibit deepwater exchange and cause prolonged residence times of the water masses, which lead to periods of oxygen depletion [65], but this may also contribute to the isolated position of the newly described new taxon. A similar example is the recent report of a relict population of the arctic nudibranch *Dendronotus velifer* in the Swedish Gullmar fjord [66].

The nudibranch fauna of the Scandinavian fjords is one of the best studied faunas in the world; however, despite an intensive search in various areas of Norway and Sweden the new genus and species *Bohuslania matsmichaeli* has been found only within single fjord, the Idefjord, and moreover in some very limited areas of the internal parts of this fjord. Therefore, it is unlikely that such a very restricted distribution of *Bohuslania* is due to insufficient data from other fjords. Instead, such a unique distribution could imply a particular historical pattern of formation of this species within the larger Scandinavian-Baltic region which has had a very convoluted history of dynamic interaction between marine and freshwater basins.

### Brackish water-diversification triggered by paedomorphosis-driven evolution

Paedomorphosis is an important evolutionary driving force [67–69], although the importance for taxonomy and phylogenetics may be considerably underestimated [70–74]. The present molecular phylogenetic analysis (Figs 1 and 3) shows that the well-known predominantly brackish-water cosmopolitan aeolidacean species *Tenellia adspersa* and the hereby described exclusively brackish-water living, but very restricted, endemic species *Bohuslania matsmichaeli* are placed in completely separated phylogenetic clades,and belong to different aeolidacean families; Trinchesiidae and Cuthonidae respectively (Fig 1). The presence of an oral veil instead of the usual oral tentacles within the derived (according to the molecular data in [8]; present study) genus *Tenellia* s. str. is a clear sign for secondary regaining of this character since the oral veil is a plesiomorphic state for the whole of the Nudibranchia [7]. Because a small oral veil is part of early ontogeny of various non-directly related aeolidaceans [26, 75] and transforms into oral tentacles only in a later developmental stage, it is a very good example of pseudoplesiomorphy caused by paedomorphosis-like ontogenetic shifts [74].

This adds a very special perspective for the present study, since the newly described genus *Bohuslania* demonstrates several features which are consistent with those found in late juveniles of 5–10 mm length of the species of the sister genus *Cuthona*, i.e. 3–4 rows of anterior cerata, absence of pre-rhinophoral ceratal rows, less prominent lateral lobes of head [12,76, present study]. Adult mature *Cuthona nana* may on the contrary have up to 10 anterior ceratal rows, well defined pre-rhinophoral ceratal rows and lateral head expansion [12,76, present study, Fig 4]. Thus, mature specimens of *B*. *matsmichaeli* correspond to the late juvenile specimens of *C*. *nana* by several characters, and also by body size (length of adult *B*. *matsmichaeli* does not exceed 10 mm, whereas adult *C*. *nana* may reach 30 mm in length). This could imply that the common ancestor of *Cuthona* and *Bohuslania* was more similar to *Cuthona* in the adult stages, but that in the *Bohuslania* line a heterochronic process of juvenilization occurred. There is another very interesting implication of the heterochrony-driven speciation in *Bohuslania*. It was previously specifically concluded [76] that the original description of the type species of the genus, *Cuthona*, *C*. *nana* was originally based on immature specimens. This generated a long-term taxonomic confusion that was finally settled only relatively recently [12, 49]. By the discovery of *Bohuslania* we thus for the first time have shown the real existence of adult mature specimens of a member of the family Cuthonidae with juvenile features that are morphologically similar to previously discussed immature late juvenile features of *C*. *nana* and further confirm this with molecular phylogenetic data. *Bohuslania matsmichaeli* is proven to be fully mature and able to reproduce at the size no more than 10 mm, which corresponds to the immature stages of *C*. *nana* (Fig 4).

The present molecular phylogenetic analysis (Fig 1) as well as a recently published major reassessment of the of the aeolidacean nudibranchs [9] shows that a reduction of the digestive gland branches has occurred in most of the genera of the family Trinchesiidae, and hence also can be connected to developmental-driven heterochronic changes. However, even among the generally more paedomorphic family Trinchesiidae the predominantly brackish-water genus *Tenellia* s.str. is the only genus in which such an earlier juvenile feature as a secondary oral veil has appeared, instead of oral tentacles (Fig 4). All other genera of the family Trinchesiidae invariably have well defined oral tentacles. In its turn, the newly discovered genus *Bohuslania* is the only genus of the family Cuthonidae that demonstrates evident persistence of the late juvenile features at adult stages (Fig 4). Thus, *Bohuslania* has acquired some paedomorphic features in parallel with *Tenellia* s. str. which belong to different molecular clades and different families (Fig 1). Since *Tenellia* and *Bohuslania* are the only predominantly brackish water-associated aeolidacean nudibranchs, this might suggest that brackish water speciation at least in the Nudibranchia can be facilitated by paedomorphosis-related heterochronic shifts in ontogeny. Another potential example of paedomorphic tendencies in the formation of an oral veil-like structure (oral tentacles still present and connected in the middle with a narrow strand, like a semi-oral veil) in *“Cuthona” rolleri* Behrens & Gosliner, 1988 [77]. This is a species living in a special environment from a geographically very distant location; mud flats in the northeastern Pacific. The combination of external morphology, radular features and details of reproductive system in *“Cuthona” rolleri* could also imply that it represents a separate genus belonging to the family Cuthonidae or to some related basal families. A species with somewhat juvenile morphology, *“Precuthona” chrysanthema* Roginskaya, 1987 was described from the White Sea [78] and was subsequently synonymized with *Cuthona nana* [79]. However, in the first description of *“P*.*” chrysanthema* the large, irregularly placed convex white spots on the cerata and yellow digestive gland were specially noted [78]. These characters correspond to a single White Sea species, *Zelentia pustulata*, according to the most recent data [7]. *Cuthona nana* and *Bohuslania matsmichaeli* never possess any big white spots on the cerata (Fig 4). On the other hand, for*“P*.*” chrysanthema* an absence of penial stylet was also mentioned [78], which is characteristic for the family Cuthonidae and not for Trinchesiidae, including *Zelentia* [7]. The putative absence of the stylet, even from a very small specimen [78], was a reason for synonymy of the latter species with *C*. *nana* in [79]. In the light of the considerable external similarity of *“P*.*” chrysanthema* to *Z*. *pustulata* and not to *C*. *nana* we therefore propose here that presence of penial stylet was not recognized for *“P*.*” chrysanthema* in [78].*“P*.*” chrysanthema* is thus considered as a synonym of *Zelentia pustulata* (= *“P*.*” chrysanthema* syn. n.). *“P*.*” chrysanthema* most likely represents a juvenile of *Z*. *pustulata* and not *C*. *nana*.

There are also additional interesting features that unite the phylogenetically unrelated, but both heterochrony-driven, genera *Tenellia* and *Bohuslania*. Both *T*. *adspersa* and *B*. *matsmichaeli* share a brownish to blackish coloration (Figs 2 and 4) in strong contrast with commonly colourful North Atlantic aeolidacean nudibranchs from areas with normal oceanic salinity. This feature needs additional investigation but together with other reduced paedomorphosis-related characters of *Tenellia* and *Bohuslania* could be related to the variability of the available nutrient content of the brackish water environment and also to the osmotic stress, that might cause the delay of development of certain characters (that was showed for example for the blue mussel *Mytilus edulis* in the Baltic Sea [80]) and indirectly produce some adaptations of these taxa to this specific niche.

When considering the paedomorphosis-driven external features, *Tenellia* and *Bohuslania* could be erroneously assessed as closely related taxa, but the present molecular analysis (Fig 1) as well as internal morphological data clearly suggest that these genera belong to different aeolidacean families (Figs 1 and 3). This is to date the first evident case of a parallel brackish-water phylogenetic diversification driven by developmental heterochronic changes.

## References

[1] Wägele H, Klussmann-Kolb A, Verbeek E, Schrödl M. Flashback and foreshadowing–a review of the taxon Opisthobranchia. Org Diver Evol. 2014; 14: 133–149. doi: 10.1007/s13127-013-0151-5

[2] Newcomb JM., Sakurai A, Lillvis JL, Gunaratne CA, Katz PS. Homology and homoplasy of swimming behaviors and neural circuits in the Nudipleura (Mollusca, Gastropoda, Opisthobranchia). PNAS. 2012; 109: 10669–10676. doi: 10.1073/pnas.1201877109 22723353 10.1073/pnas.1201877109 PMC3386871

[3] Blunt JW, Copp BR, Keyzers RA., Munro MH, Prinsep MR. Marine natural products. Nat Prod Rep. 2015; 32: 116–211. doi: 10.1039/c4np00144c . 25620233 10.1039/c4np00144c

[4] Lindsay T, Valdés Á. The model organism *Hermissenda crassicornis* (Gastropoda: Heterobranchia) is a species complex. PLoSOne. 2016; 11: e0154265 doi: 10.1371/journal.pone.0154265 27105319 10.1371/journal.pone.0154265 PMC4841509

[5] Carmona L, Pola M, Gosliner TM, Cervera JL. A tale that morphology fails to tell: A molecular phylogeny of Aeolidiidae (Aeolidida, Nudibranchia, Gastropoda). PLoSOne. 2013; 8: e63000 doi: 10.1371/journal.pone.0063000 23658794 10.1371/journal.pone.0063000 PMC3642091

[6] Goodheart JA. Insights into the systematics, phylogeny, and evolution of Cladobranchia (Gastropoda: Heterobranchia). Amer Malac Bull. 2017; 35: 73–81. doi: 10.4003/006.035.0111

[7] Korshunova T, Martynov A, Picton B. Ontogeny as an important part of integrative taxonomy in tergipedid aeolidaceans (Gastropoda: Nudibranchia) with a description of a new genus and species from the Barents Sea. Zootaxa. 2017; 4324: 1–022. doi: 10.11646/zootaxa.0000.0.0

[8] Cella K, Carmona L, Ekimova I, Chichvarkhin A, Schepetov D, Gosliner TM. A Radical Solution: The phylogeny of the nudibranch family Fionidae. PLoSOne. 2016; 11: e0167800 doi: 10.1371/journal.pone.0167800 27977703 10.1371/journal.pone.0167800 PMC5158052

[9] Korshunova T, Martynov A, Bakken T, Evertsen J, Fletcher K, Mudianta WI et al Polyphyly of the traditional family Flabellinidae affects a major group of Nudibranchia: aeolidacean taxonomic reassessment with descriptions of several new families, genera, and species (Mollusca, Gastropoda). ZooKeys 2017; 717: 1–139. 10.3897/zookeys.717.21885 PMC5784208 29391848

[10] Evertsen J, Bakken T, Green S. Rediscovery of *Tenellia adspersa* (Nudibranchia) from the Finnish archipelago. Sarsia. 2004; 89: 362–365. doi: 10.1080/00364820410002569

[11] Martynov AV, Korshunova TA, Grintsov VA. Opisthobranch molluscs of the Northern Black Sea. I. Short history of studies and the first record of a non-indigenous nudibranch species *Trinchesia perca* (Er. Marcus, 1958) (Nudibranchia: Tergipedidae). Ruthenica. 2007; 17: 43–54.

[12] Thompson TE, Brown GH. Biology of Opisthobranch Molluscs, Vol. 2. London: The Ray Society Publications, 1984.

[13] Neusser TP, Fukuda H, Jörger KM, Kano M, Schrödl M. Sacoglossa or Acochlidia? 3D reconstruction, molecular phylogeny and evolution of Aitengidae (Gastropoda: Heterobranchia). Journal of Molluscan Studies. 2011; 77 (4): 332–350. doi: 10.1093/mollus/eyr033

[14] Kano Y, Brenzinger B, Nützel A, Wilson N G, Schrödl M. Ringiculid bubble snails recovered as the sister group to sea slugs (Nudipleura). Scientific reports. 2016; 6: 30908 doi: 10.1038/srep30908 27498754 10.1038/srep30908 PMC4976385

[15] Forrestel EF, Ackerly DD, Emery NC. The joint evolution of traits and habitat: ontogenetic shifts in leaf morphology and wetland specialization in *Lasthenia*. New Phytol. 2017; 208: 949–959. doi: 10.1111/nph.13478 26037170 10.1111/nph.13478

[16] Roxio FF, Lujan NK, Tagliacollo VA, Waltz BT, Silva GSC, Oliveira C et al Shift from slow- to fast-water habitats accelerates lineage and phenotype evolution in a clade of Neotropical suckermouth catfishes (Loricariidae: Hypoptopomatinae). PLoSOne. 2017; 12: e0178240 doi: 10.1371/journal.pone.0178240 28591189 10.1371/journal.pone.0178240 PMC5462362

[17] Schoener TW. Competition and the form of habitat shift. Theor Popul Biol. 1974; 6: 265–307. 4460260 10.1016/0040-5809(74)90013-6

[18] Vitt LJ, Caldwell JP, Zani PA, Titus TA. The role of habitat shift in the evolution of lizard morphology: evidence from tropical *Tropidurus*. PNAS. 1997; 94: 3828–3832. PMCID: PMC20526 9108063 10.1073/pnas.94.8.3828 PMC20526

[19] Cognetti G, Maltagliati F. Biodiversity and adaptive mechanisms in brackish water fauna. Mar Poll Bull. 2000; 40: 7–14. doi: 10.1016/S0025-326X(99)00173-3

[20] Delicado D, Machordom A, Ramos MA. Effects of habitat transition on the evolutionary patterns of the microgastropod genus *Pseudamnicola* (Mollusca, Hydrobiidae). Zool Scripta. 2015; 44: 403–417. doi: 10.1111/zsc.12104

[21] Bilton DT, Paula J, Bishop JDD. Dispersal, genetic differentiation and speciation in estuarine organisms. Estuar Coast Shelf Scie. 2015; 55: 937–952. doi: 10.1006/ecss.2002.1037

[22] Whitfield AK, Elliott M, Basset A. Blaber SJM, West RJ. Paradigms in estuarine ecology: A review of the Remane diagram with a suggested revised model for estuaries. Estuar Coast Shelf Scie. 2012; 97: 78–90. doi: 10.1016/j.ecss.2011.11.026

[23] Ashelby CW, Page TJ, De Grave S, Hughes JM, Johnson ML. Regional scale speciation reveals multiple invasions of freshwater in Palaemoninae (Decapoda). Zool Scripta. 2012; 41: 293–306. doi: 10.1111/j.1463-6409.2012.00535.x

[24] Boeger WA, Marteleto FM, Zagonel L, Braga MP. Tracking the history of an invasion: the freshwater croakers (Teleostei: Sciaenidae) in South America. Zool Scripta. 2015; 44: 250–262. doi: 10.1111/zsc.12098

[25] Jörger KM, Brenzinger B, Neusser TP, Martynov AV, Nerida G. Wilson et al Panpulmonate habitat transitions: tracing the evolution of Acochlidia (Heterobranchia, Gastropoda). Bioarxiv. 2014; https://doi.org/10.1101/010322

[26] Eyster LS. Reproduction and developmental variability in the opisthobranch *Tenellia pallida*. Mar. Biol. 1979; 51: 133–140. doi: 10.1007/BF00555192

[27] Neusser TP, Schrödl M. Between Vanuatu tides: 3D anatomical reconstruction of a new brackish water acochlidian gastropod from Espiritu Santo. Zoosystema. 2009; 31: 453–469. doi: 10.5252/z2009n3a4

[28] Stenström A. Marinbiologisk undersökning i de inre delarna av Idefjorden 2010. The County Administrative Board of Västra Götaland, report 2010–65, 2010.

[29] Hariyama T, Takaku Y. Dressing living organisms in the NanoSuit® for FE-SEM observation. JEOL NEWS. 2015; 50: 26–37.

[30] Takaku Y, Suzuki H, Ohta I, Tsutsui T, Matsumoto H, Shimomura M, et al 'NanoSuit' surface shield successfully protects organisms in high vacuum: observations on living organisms in an FE-SEM. Proc Biol Sci. 2015; 282: 20142857 doi: 10.1098/rspb.2014.2857 25631998 10.1098/rspb.2014.2857 PMC4344158

[31] Martynov AV, Sanamyan NP, Korshunova TA. New data on the opisthobranch molluscs (Gastropoda: Opisthobranchia) of waters of Commander Islands and Far-Eastern seas of Russia. Conservation of biodiversity of Kamchatka and coastal waters. Proceedings of XV international scientific conference Petropavlovsk-Kamchatsky. Kamchat Press, Petropavlovsk-Kamchatsky, 2015.

[32] Martynov AV, Sanamyan NP, Korshunova TA. Review of the opisthobranch mollusc fauna of Russian Far Eastern seas: Pleurobranchomorpha, Doridida and Nudibranchia. Bull Kamch State Techn Univ. 2015; 34: 62–87. doi: 10.17217/2079-0333-2015-34-62-87

[33] Chichvarkhin AY, Ekimova I, Egorova E, Chichvarkhina OV. Species identity of a nudibranch mollusk of the genus *Cuthona* associated with hermit crabs in the sea of Japan. 2016; Biol Morya; 42: 449–457.

[34] Edgar RC. MUSCLE: Multiple sequence alignment with high accuracy and high throughput. Nucleic Acids Res. 2004; 32: 1792–1797. doi: 10.1093/nar/gkh340 15034147 10.1093/nar/gkh340 PMC390337

[35] Talavera G, Castresana J. Improvement of phylogenies after removing divergent and ambiguosly aligned blocks from protein sequence alignments. Syst. Biol. 2007; 56: 564–577. doi: 10.1080/10635150701472164 17654362 10.1080/10635150701472164

[36] Nylander JA, Ronquist F, Huelsenbeck JP, Nieves-Aldrey JL. Bayesian phylogenetic analysis of combined data. Syst Biol. 2004; 53: 47–67. 14965900 10.1080/10635150490264699

[37] Akaike H. A new look at the statistical model identification. IEEE Trans Automat Control. 1974; 19: 716–723. doi: 10.1007/978-1-4612-1694-0_16

[38] Ronquist F, Teslenko M, van der Mark P, Ayres DL, Darling A, Höhna S et al MrBayes 3.2: Efficient Bayesian phylogenetic inference and model choice across a large model space. Syst. Biol. 2012; 61: 539–542. doi: 10.1093/sysbio/sys029 22357727 10.1093/sysbio/sys029 PMC3329765

[39] Stamatakis A, Hoover P, Rougemont J. A Rapid bootstrap algorithm for the RAxML web-servers. Syst. Biol. 2008; 75: 758–771. doi: 10.1080/10635150802429642 18853362 10.1080/10635150802429642

[40] Kumar S, Stecher G, Tamura K. MEGA7: Molecular evolutionary genetics analysis version 7.0 for bigger datasets. Mol Biol Evol. 2016; 33: 1870–1874. doi: 10.1093/molbev/msw054 27004904 10.1093/molbev/msw054 PMC8210823

[41] Puillandre N, Lambert A, Brouillet S, Achaz G. ABGD, Automatic barcode gap discovery for primary species delimitation. Mol Ecol. 2012; 21: 1864–1877. doi: 10.1111/j.1365-294X.2011.05239.x 21883587 10.1111/j.1365-294X.2011.05239.x

[42] Dayrat B. Towards integrative taxonomy. Biol J Linn Soc. 2005; 85, 407–415. doi: 10.1111/j.1095-8312.2005.00503.x

[43] Carmona L, Pola M, Gosliner TM, Cervera JL. The Atlantic-Mediterranean genus *Berghia* Trinchese, 1877 (Nudibranchia: Aeolidiidae): taxonomic review and phylogenetic analysis. J Moll Stud. 2014; 80: 482–498. doi: 10.1093/mollus/eyu031

[44] Kienberger K, Carmona L, Pola M, Padula V, Gosliner TM, Cervera JL. *Aeolidia papillosa* (Linnaeus, 1761) (Mollusca: Heterobranchia: Nudibranchia), single species or a cryptic species complex? A morphological and molecular study. Zool J Linn Soc. 2016; 177: 481–506. doi: 10.1111/zoj.12379

[45] Korshunova T, Zimina O, Martynov A. Unique pleuroproctic taxa of the nudibranch family Aeolidiidae from the Atlantic and Pacific Oceans, with description of a new genus and species. J Moll Stud. 2017 doi: 10.1093/mollus/eyx036

[46] Martin R, Heß M, Schrödl M, Tomaschko KH. Cnidosac morphology in dendronotacean and aeolidacean nudibranch molluscs: from expulsion of nematocysts to use in defense? Mar Biol. 2009; 156: 261–268. doi: 10.1007/s00227-008-1080-2

[47] Martin R, Tomaschko KH, Heß M, Schrödl M. Cnidosac-related structures in *Embletonia* (Mollusca, Nudibranchia) compared with Dendronotacean and Aeolidacean species. Open Mar Biol J. 2010; 4: 96–100. doi: 10.2174/1874450801004010096

[48] Martynov AV. A new species of nudibranch molluscs of the Sea of Japan with notes on the genus *Cuthonella* (Gastropoda, Opisthobranchia). Zool Zhurn. 1992; 71: 18–24.

[49] Martynov AV. Two new species of the genus *Trinchesia* Ihering, 1879 from Peter the Great Bay, Japan Sea (Nudibranchia, Tergipedidae), with notes on the taxonomy of the family. Ruthenica. 2002; 12: 45–54.

[50] Miller MC. An appraisal of the identity of the New Zealand species of the aeolid nudibranch family Tergipedidae (Gastropoda: Opisthobranchia). J Nat Hist. 2004; 38: 1183–1192. doi: 10.1080/0022293031000077699

[51] Miller MC, Willan RC. A redescription and taxonomic reappraisal of the Indo-Pacific aeolid nudibranch *Trinchesia sibogae* (Bergh, 1905) (Gastropoda, Opisthobranchia). Vita Malacologica. 2005; 3: 69–75.

[52] Bonsdorff E. Zoobenthic diversity-gradients in the Baltic Sea: Continuous post-glacial succession in a stressed ecosystem. J Exp Mar Biol Ecol. 2006; 330: 383–391. doi: 10.1016/j.jembe.2005.12.041

[53] Baltazar-Soares M, Paiva F, Chen Y, Zhan A. Diversity and distribution of genetic variation in gammarids: Comparing patterns between invasive and non-invasive species. Ecol Evol. 2017; 1–12. doi: 10.1002/ece3.3208 29043025 10.1002/ece3.3208 PMC5632605

[54] Dolmen D, Hindley JD, Kleiven E. Distribution of *Palaemonetes varians* (Leach) (Crustacea, Decapoda) in relation to biotope and other caridean shrimps in brackish waters of southern Norway and southwestern Sweden. Sarsia. 2004; 89: 8–21. doi: 10.1080/00364820310003244

[55] Norenburg J. Redescription of a brooding nemertine, *Cyanophthalma obscura* (Schultze) gen. et. comb.n., with observations on its biology and discussion of the species of *Prostomatella* and related taxa. Zool. Scripta. 1986; 15: 227–293. doi: 10.1111/j.1463-6409.1986.tb00229.x

[56] Scaps P. A review of the biology, ecology and potential use of the common ragworm *Hediste diversicolor* (O.F. Müller) (Annelida: Polychaeta). Hydrobiologia. 2002; 470, 203–218. doi: 10.1023/A:1015681605656

[57] Meek MH, Wintzer AP, Wetzel WC, May B. Climate change likely to facilitate the invasion of the non-native hydroid, *Cordylophora caspia*, in the San Francisco Estuary. PLoSONE. 2012; 7: e46373 doi: 10.1371/journal.pone.0046373 23071559 10.1371/journal.pone.0046373 PMC3469613

[58] Mees J, Abdulkerim Z, Hamerlynck O. Life history, growth and production of *Neomysis integer* in the Westerschelde estuary (SW Netherlands). Mar Ecol Progr Ser. 1994; 109: 43–57. doi: 10.3354/meps109043

[59] Smith DG, Smith SF, Werle E, Klekowski SF. The rapid colonization and emerging biology of *Cordylophora caspia* (Pallas, 1771) (Cnidaria: Clavidae) in the Connecticut River. J Freshw Ecol. 2002; 17: 423–430.

[60] Steele DH, Steele VJ. The biology of *Gammarus* (Crustacea, Amphipoda) in the northwestern Atlantic. I. *Gammarus duebeni* Lillj. Canad J Zool. 1969; 47: 235–244. doi: 10.1139/z69-050

[61] Grabowska M., Kukliński P. Spatial pattern of hydrolittoral rock encrusting assemblages along the salinity gradient of the Baltic Sea. Hydrobiologia. 2016; 765: 297–315. doi: 10.1007/s10750-015-2421-z

[62] Carlsson R. The distribution of the gastropods Theodoxus fluviatilis (L.) and Potamopyrgus antipodarum (Gray) in lakes on the Aland Islands, southwestern Finland. Boreal environment research. 2000; 5: 187–195.

[63] Zettler ML, Bick A, Bochert R. Distribution and population dynamics of *Marenzelleria viridis* (Polychaeta, Spionidae) in a coastal water of the southern Baltic. Arch Fishery Mar Res. 1995; 42: 209–224.

[64] Roginskaya IS. *Tenellia adspersa*, a nudibranch new to the Azov Sea, with notes on its taxonomy and ecology. Malacol Rev. 1970; 3: 167–174.

[65] Davidsson S, Martinsson S, Gomes W. The Recovery- pollution and chronology in the Idefjord located on the Swedish west coast, on the border between Sweden and Norway. Göteborg: Department of Earth sciences, University of Gothenburg C115, 2015.

[66] Lundin K, Korshunova T, Malmberg K, Martynov A. Intersection of historical museum collections and modern systematics: a relict population of the Arctic nudibranch *Dendronotus velifer* G.O. Sars, 1878 in a Swedish fjord. Contr Zool. 2017; 86: 303–318.

[67] Bhullar BA, Marugán-Lobón J, Racimo F, Bever GS, Rowe TB, Norell MA, et al Birds have paedomorphic dinosaur skulls. Nature. 2012; 487: 223–226. doi: 10.1038/nature11146 22722850 10.1038/nature11146

[68] Härer AH, Torres-Dowdall J, Meyer A. Rapid adaptation to a novel light environment: the importance of ontogeny and phenotypic plasticity in shaping the visual system of Nicaraguan Midas cichlid fish (*Amphilophus citrinellus* spp.). Mol Ecol. 2017 doi: 10.1111/mec.14289 28792657 10.1111/mec.14289

[69] Martynov A, Schrödl M. Phylogeny and evolution of corambid nudibranchs (Mollusca: Gastropoda). Zool J Linn Soc. 2011; 163: 585–604. doi: 10.1111/j.1096-3642.2011.00720.x

[70] Kerbl A, Fofanova EG, Mayorova TD, Voronezhskaya EE, Worsaae K. Comparison of neuromuscular development in two dinophilid species (Annelida) suggests progenetic origin of *Dinophilus gyrociliatus*. Front Zool. 2016; 13: 49 doi: 10.1186/s12983-016-0181-x 27833644 10.1186/s12983-016-0181-x PMC5101659

[71] Oromi N, Michaux J, Denoël M. High gene flow between alternative morphs and the evolutionary persistence of facultative paedomorphosis. Sci. Rep. 2016; 6: 32046 doi: 10.1038/srep32046 27534370 10.1038/srep32046 PMC4989185

[72] Bocak L, Kundata R, Andujar Fernandez C, Vogler AP. The discovery of Iberobaeniidae (Coleoptera: Elateroidea): a new family of beetles from Spain, with immatures detected by environmental DNA sequencing. Proceedings of the Royal Society B. 2016; 283: 20152350 doi: 10.1098/rspb.2015.2350 27147093 10.1098/rspb.2015.2350 PMC4874698

[73] Barfod AS. Letter to the twenty-first century botanist–what is a flower? 4. Heterochrony–still an overlooked source of rapid morphological change in flowers? Bot Lett. 2017; 164: 105–109. doi: 10.1080/23818107.2017.1314830

[74] Stöhr S, Martynov A. Paedomorphosis as an evolutionary driving force: Insights from Deep-Sea brittle stars. PLoSOnE. 2016 11: e0164562 doi: 10.1371/journal.pone.0164562 27806039 10.1371/journal.pone.0164562 PMC5091845

[75] Tardy JP. Contribution a l'étude des métamorphoses chez les nudibranches. Ann Sci Naturel. 1970; 12: 299–370.

[76] Brown GH. The British species of the aeolidacean family Tergipedidae (Gastropoda: Opisthobranchia) with a discussion of the genera. Zool J Linn Soc. 1980; 69: 225–255. doi: 10.1111/j.1096-3642.1980.tb01124.x

[77] Behrens DW, Gosliner TM. A new species of tergipedid nudibranch from Morro Bay, California. The Veliger. 1988; 31: 262–266.

[78] Roginskaya IS. Order Nudibranchia Blainville, 1814 In: Molluscs of the White Sea. Keys to Fauna of SSSR, ZIN AN SSSR, 1987.

[79] Martynov AV. Nudipleura. In: Kantor Yu.I., Sysoev A.V. (Eds.), Marine and brackish water Gastropoda of Russia and adjacent countries: an illustrated catalogue. Moscow: KMK Scientific Press Ltd, 2006.

[80] Westerbom M., Kilpi M., Mustonen O. Blue mussels, *Mytilus edulis*, at the edge of the range: population structure, growth and biomass along a salinity gradient in the north-eastern Baltic Sea. Mar Biol, 2002; 140: 991–999.

[81] Folmer O, Black M, Hoeh W, Lutz R, Vrijenhoek R. DNA primers for amplification of mitochondrial cytochrome c oxidase subunit I from diverse metazoan invertebrates. Molecular Mar Biol Biotech. 1994; 3: 294–299. 7881515

[82] Palumbi SR, Martin AP, Romano S, McMillan WO, Stice L, Grabowski G. The simple fool’s guide to PCR. Honolulu: University of Hawaii, 2002.

[83] Puslednik L, Serb JM. Molecular phylogenetics of the Pectinidae (Mollusca: Bivalvia) and effect of increased taxon sampling and outgroup selection on tree topology. Mol Phyl Evol. 2008; 48: 1178–1188. doi: 10.1016/j.ympev.2008.05.006 18579415 10.1016/j.ympev.2008.05.006

[84] Colgan D, Macaranas J, Cassis G, Gray MR. Histone H3 and U2 snRNA DNA sequences and arthropod molecular evolution. Austr J Zool. 1998; 4: 419–437.

[85] Dayrat B, Tillier A, Lecointre G, Tillier S. New clades of euthyneuran gastropods (Mollusca) from 28S rRNA sequences. Mol Phyl Evol. 2001; 19: 225–235. 10.1006/mpev.2001.0926 11341805

[86] Le HLV, Lecointre G, Perasso R. A 28S rRNA based phylogeny of the Gnathostomes: First steps in the analysis of conflict and congruence with morphologically based cladograms. Mol Phyl Evol, 1993; 2: 31–51. 10.1006/mpev.1993.1005 8081546

